# Benefit, Necessity or Harm by Administering Heparin during Neurointerventional Procedures?

**DOI:** 10.1007/s00270-021-02794-5

**Published:** 2021-03-11

**Authors:** Ansgar Berlis

**Affiliations:** Diagnostic and Interventional Neuroradiology, University Hospital Augsburg, Augsburg, Germany

Intraarterial or intravenous heparinization during neurointerventional procedures has been used regularly for over 30 years. Heparin is administered either as a single bolus, or continuously intravenously, and/or continuously via saline flushing of the catheter. Thromboembolism was and is until today the most common complication resulting in neurological deficits during neurointerventions. The technical advances and the continuously increasing number of neurointerventions have significantly shortened the procedures’ duration. Interventions that last many hours belong to the past and have become very rare. Additionally, to the benefit of time reduction, managing of anticoagulation and antiaggregation treatment has improved. As a result, many elective interventions are performed under controlled platelet inhibition. Furthermore, emergency treated patients are not seldom taking anticoagulants or antiplatelets. Therefore, the role of intraprocedural heparin application must be reconsidered—not least under the aspect that there are no standards for heparinization.

Benaly et al. [1] analyzed the Mr Clean data with regards to the effect of heparin on bleeding rates and outcome. Of course, there are no national or international recommendations regarding the use of heparinized flushing fluid during endovascular interventions and especially in thrombectomy (EVT) for acute ischemic stroke (AIS).

Therefore, the manuscript raises a very important question:How much heparin is necessary and how much heparin is dangerous? The data analysis only shows a trend without a level of significance. Nevertheless, it is interesting that the group with 25.000 heparin units stands out in comparison to the other groups in the following points: second lowest recanalization rate, highest mortality rate, highest rate of using stent retriever, fastest management with onset to groin puncture, longest procedure time with probably the highest amount of heparinized infusion, highest rate of previous anticoagulated patients. The influence of all these factors certainly determines the results.

However, this is an important observational study that clearly shows that little or no administration of heparin does not lead to more complications and, as a trend, to less symptomatic bleeding.

What can we learn for our daily practice? Flushing of the catheter systems might be more important than using heparin in the flushing fluid, especially in EVT and additional use of fibrinolytics. The main problem, however, is that when Heparin is administered via saline flushing, there is no adequate dose monitoring of the heparin effect. Our own daily practice does not include heparin in saline flushing, neither in elective nor in emergency procedures. Heparin is usually administered as a bolus of 5.000 units, but not in stroke patients with EVT and not if the expected duration of the procedure is less than 60 min. However, in case of venous interventions heparinization is always used. Keep in mind that thromboembolic complications are treated very effectively with intravenously application of GP IIb/IIIa inhibitors.

Is there a need for a randomized trial to get evidence? Is there a need for standardization? The first question cannot be answered because heparin administration without a clear dose plan versus no heparin cannot be examined. The second question seems to be easier at first glance. However, standards also mean that failure to comply could have legal consequences.

My personal recommendation is: The administration of heparin during neurointerventional procedures should be weighed against a specific risk of bleeding. This includes accompanying medication, duration and type of procedure. Heparin should be avoided in patients with anticoagulants or fibrinolytic treatment. When heparin is administered, the exact amount must be documented. As always, it is a personal experience from a high volume center and there is a lack of scientific evidence.

## References

[1] Benali F, Hinsenveld WH, van der Leij C (2021). Effect of Heparinized Flush Concentration on Safety and Efficacy During Endovascular Thrombectomy for Acute Ischemic Stroke: Results from the MR CLEAN Registry. Cardiovasc Intervent Radiol.

